# Restricting Admissions to Poor-Law Infirmaries

**Published:** 1915-03-27

**Authors:** 


### RESTRICTING ADMISSIONS TO POOR-LAW INFIRMARIES.

An inquest recently held at Rustington brings again into prominence the dangers which may arise from the views held by many Boards of Guardians that admission to Poor-Law infirmaries should, as a rule, be restricted to those sick who are also destitute. This opinion is still frequently expressed at Guardians' meetings all over the country, despite the circular-letter of the Local Government Board of March 18, 1910, pointing out that "a person who is not destitute in the sense that he is entirely devoid of the means of subsistence may yet be destitute in that he is unable to provide for himself the particular form of medical attendance or treatment of which he is in urgent need." Some Boards have gone so far as to pass resolutions either directly limiting the power of the relieving officer to issue orders of admission to the infirmary or suggesting that orders should be given only in cases of extreme urgency. Such resolutions do not absolve the relieving officer from the responsibilities placed upon him by the Local Government Board Orders, but they put him in a very difficult position.

The Littlehampton Guardians on February 9 of this year instructed their relieving officers not to give orders for the workhouse infirmary before the meeting of the Board, except in urgent cases of sick people who were also destitute, and that in each case an explanatory letter from the medical officer in charge of the case was to be produced at the next meeting. The evil consequences of this resolution were not long in materialising. On February 25 Dr. Chaplin, who was attending a case of septic meningitis in Rustington, in the Littlehampton Union, advised the mother to apply for the removal of the patient to the workhouse infirmary. The case was seen by Dr. Last, the district medical officer, who gave the necessary certificate for the removal. Upon receiving this recommendation the relieving officer saw Dr. Last, drew his attention to the Guardians' resolution, and asked him if he considered it an urgent case. To this Dr. Last replied that he thought the case would die whatever was done, and, that being so, he could not say the removal was a matter of urgent necessity. Questioned by the Coroner, he stated that the nursing the case was likely to receive from her mother, who was working as a laundress, was not such as she would receive at the infirmary, and it was upon that ground that he made the recommendation for admission. The relieving officer, however, decided not to remove the case until he had referred it to the Guardians, who did not meet until twelve days later. The patient died on February 28. Dr. Chaplin, addressing the Coroner, said that he made no attack on the relieving officer, who, he believed, acted under the Guardians' instruction, but that he hoped that a public inquiry might make it plain to the Guardians that they were responsible for the poor of the district, who were as much entitled to infirmary treatment as the workhouse inmates, and that it was the duty of the Guardians to assure admission of acute cases without delay. This statement was applauded by the jury, and was embodied in a rider to their verdict.

The case is of great importance, because so many Guardians hold views similar to those which rule at Littlehampton. A motion almost identical in its terms with that adopted by the Littlehampton Guardians was discussed recently by the East Preston Guardians, and was only defeated by eleven votes to nine. It is difficult to speak temperately of a public authority which takes up an attitude of this kind. It shows clearly the urgent necessity for a drastic revision of our Poor-Law system. The evidence placed before the Royal Commission on the Poor Laws proved conclusively the importance of the prompt provision of medical treatment, and one of the recommendations accepted by all the Commissioners was that in cases of sickness the physical condition of the applicant should be the first consideration. With this recommendation the great majority of the community are in hearty sympathy, while it is depressing to find many Boards of Guardians still lagging behind.

